# Modulation of Blood–Brain Barrier Permeability by Activating Adenosine A2 Receptors in Oncological Treatment

**DOI:** 10.3390/biom11050633

**Published:** 2021-04-24

**Authors:** Kamila Wala, Wojciech Szlasa, Jolanta Saczko, Julia Rudno-Rudzińska, Julita Kulbacka

**Affiliations:** 1 Faculty of Medicine, Wroclaw Medical University, Pasteura 1, 50-367 Wroclaw, Poland; kamila.wala@outlook.com (K.W.); wojciech.szlasa@outlook.com (W.S.); 2 Department of Molecular and Cellular Biology, Faculty of Pharmacy, Wroclaw Medical University, Borowska 211A, 50-556 Wroclaw, Poland; jolanta.saczko@umed.wroc.pl; 3 Department of General and Oncological Surgery, Medical University Hospital, Borowska 213, 50-556 Wrocław, Poland; julia.rudno-rudzinska@umed.wroc.pl

**Keywords:** blood–brain barrier permeability, adenosine A2 receptor, A2AR agonists

## Abstract

The blood–brain barrier (BBB) plays an important protective role in the central nervous system and maintains its homeostasis. It regulates transport into brain tissue and protects neurons against the toxic effects of substances circulating in the blood. However, in the case of neurological diseases or primary brain tumors, i.e., gliomas, the higher permeability of the blood-derived substances in the brain tissue is necessary. Currently applied methods of treatment for the primary brain neoplasms include surgical removal of the tumor, radiation therapy, and chemotherapy. Despite the abovementioned treatment methods, the prognosis of primary brain tumors remains bad. Moreover, chemotherapy options seem to be limited due to low drug penetration into the cancerous tissue. Modulation of the blood–brain barrier permeability may contribute to an increase in the concentration of the drug in the CNS and thus increase the effectiveness of therapy. Interestingly, endothelial cells in cerebral vessels are characterized by the presence of adenosine 2A receptors (A2AR). It has been shown that substances affecting these receptors regulate the permeability of the BBB. The mechanism of increasing the BBB permeability by A2AR agonists is the actin-cytoskeletal reorganization and acting on the tight junctions. In this case, the A2AR seems to be a promising therapy target. This article aims to assess the possibility of increasing the BBB permeability through A2AR agonists to increase the effectiveness of chemotherapy and to improve the results of cancer therapy.

## 1. Introduction

The blood–brain barrier (BBB) plays an important protective role for the central nervous system and maintains its homeostasis. The main role of the BBB is to regulate the transport of molecules into brain tissue to maintain the right amount of nutrients for the proper functioning of neurons. At the same time, it limits the access of endo- and exo-genous metabolites, protecting cells from the toxic effects of these substances. Despite the benefits of protective function, the BBB is an obstacle for the therapeutic substances to the entry into the central nervous system [1]. A higher level of chemicals in the brain tissue is necessary to obtain a response to therapy in neurological diseases and primary brain tumors, such as gliomas. The increase in the concentration of the drug in the serum may contribute to greater availability in the CNS; however, it might also cause severe side effects. Modulation of the BBB permeability may contribute to an increase in drug concentration in the CNS, enhance the selectivity of drug delivery to the tumor area, and thus increase the effectiveness of the therapy [2].

This short review aims to assess the possibility of modulating the BBB permeability by the activation of the adenosine receptors. The review includes examples of adenosine agonists and their characterization, with particular reference to the A2 receptor agonist. The available preclinical and clinical studies on the possibility of using A2AR activators in the oncological treatment of malignant brain tumors were analyzed. Furthermore, an attempt was made to identify the mechanisms responsible for changing the permeability of the barrier in order to increase the effectiveness of oncological therapy. Despite the scant amount of in vivo and clinical trials, the authors made an effort to evaluate the clinical usefulness of adenosine A2A receptor agonists in a cotreatment of brain tumors.

## 2. Blood–Brain Barrier

### 2.1. Structure and Function

**Figure 1:**
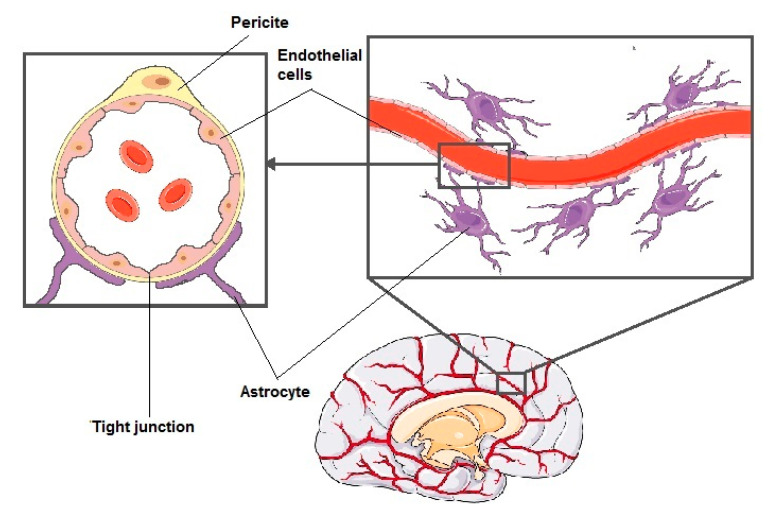
Structure of blood–brain barrier—composed of pericytes, endothelial cells, and astrocytes.

The structure of the BBB is composed of specialized endothelial cells lining small vessels in the brain, surrounded by microglia cells—astrocytes and pericytes (Figure 1). The principal component of the barrier between the blood and the CNS is the endothelium, characterized by low permeability and the presence of specific transport systems, such as P-glycoprotein, multidrug-resistance-associated proteins or breast-cancer-resistant proteins. This drug efflux system is responsible for removing, among others, vinblastine, vincristine, taxanes, and anthracyclines from the cells [3]. Modified endothelial cells also have a reduced number of transcytotic vesicles, no fenestration, and a higher electrical resistance due to tight junctions compared to typical endothelial cells [4].

Tight junctions are the protein complexes that strengthen the intercellular barrier, limit transport along the paracellular pathway, and prevent leakage of transported solutes. The junctions play a role in the regulation of the permeability of molecules and ions through the space between adjacent cells, which is essential for the proper blood–brain barrier function. Molecules that affect the integrity of the junctional complex alter the permeability of the membrane and may potentially increase the drug concentration in the CNS [4,5]. Tight junctions (TJs) are a very heterogeneous system composed of different transmembrane and cytoplasmic proteins. The transmembrane proteins include claudins, occludin, and junctional adhesion molecules (JAMs). The junction complexes are tightly attached to the actin cytoskeleton by membrane-associated proteins located on the inner side of the cytoplasmic membrane, e.g., the ZO-1 protein, causing adjacent cells to form a uniform barrier [6,7].

Astrocytes are glial cells that form a framework for the other CNS cells and surround the microvessels with their branched cell processes [8]. Another cellular component that forms the BBB are pericytes, which limit immune cell entry to the CNS, as well as regulate blood flow in cerebral microcirculation. Pericytes and endothelial cells share a mutual basement membrane that contains various proteins (e.g., integrins) involved in the formation of connections between cells [9]. 

Astrocytes together with brain capillaries and pericytes form functional ‘neurovascular units’, which support the blood–brain barrier and control the flow of water, ionic, amino acid, and neurotransmitter to the brain. The cells interact with each other by secreting several factors. For instance, glial cells affect endothelial cells by determining their phenotype, pericytes induce angiogenesis, and endothelium induces growth and differentiation of astrocytes. This causes the blood–brain barrier to be constantly regulated and dynamically modulated by physiological factors, as well as pathological ones (such as inflammation) [10]. 

### 2.2. BBB in Pathological Conditions

Various CNS pathological conditions such as stroke, inflammation, multiple sclerosis, or degenerative diseases lead to a reduction in the expression of TJ proteins. The structure and function of the blood–brain barrier are impaired, which increases the permeability of the BBB [7,11]. Malignant brain tumors such as gliomas are known to disrupt the BBB and change vascular permeability [12]. In T1-weighted MRI imaging, the contrast agent reaches the tumor parenchyma in areas with increased permeability of cerebral capillaries [13]. However, it has been proven that the cancer cells are also outside the contrast-enhanced regions [14]. Gao et al. also proved that expression of the ZO-1 proteins involved in TJ formation and ensuring BBB integrity remained partially intact at the margins of the glioma [15]. This supports the assumption that these tumors also have clinically significant areas of the intact BBB, especially at the edges of the infiltrating tumor, which prevents drug delivery to all tumor cells and contributes to the failure of treatment [2,16]. 

Through the interactions of the BBB-creating cells, the repair of this damage occurs. Namely, the cells regulate the expression of certain proteins to limit the pathological process and restore normal barrier function [10]. One of these overexpressed proteins is the A2A receptor. Its increased expression was detected in high-grade glioma vasculature, while in peritumor nonpathological brain tissue remained unaltered [15]. The knowledge about changes in the expression of certain proteins in the cerebral vascular endothelium in CNS pathologies may be used as a potential therapeutic target in the treatment of malignant brain tumors. 

## 3. Brain Tumors and the Role of the Blood–Brain Barrier in Oncological Treatment

Brain neoplasm include primary brain tumors (such as gliomas, lymphomas, germ cell tumors, or saddle tumors), as well as metastatic tumors. Treatment of brain tumors includes surgery, radiation therapy, chemotherapy, and supportive therapy. Despite many methods of treatment, the relative 5 year survival is 32.6% and has remained constant and low, compared to other cancers, for about 10 years [17]. In the case of glioblastoma multiforme, the most severe form of glioblastoma, the average patient survival is 15 months, where only 4–5% of patients survive 5 years from diagnosis [18,19]. Due to their location, malignant brain tumors pose a great therapeutic challenge, especially high-grade gliomas. Surgical resection of malignant brain tumors is recommended whenever possible, but the extent of the procedure is often limited by the high risk of damaging important functions of the central nervous system [20]. Systemic chemotherapy (e.g., with carmustine or temozolomide) is therefore standard adjuvant therapy [21]. However, only small molecules, drugs with molecular weights up to 400 Daltons, can pass through the BBB by free diffusion [22]. The other molecules have a lower concentration in the CNS, which reduces their effectiveness. However, it should be taken into account that increasing the dose of the drug is associated with more intense systemic side effects. Table 1 summarizes the BBB permeability and side effects of the chemotherapy agents used in brain tumors’ chemotherapy. 

Temozolomide, which is effective in improving the survival of patients with glioblastoma, reaches concentrations five times lower in the CNS than in the blood [48]. The decreased concentration of antineoplastic drugs in the CNS is due to the limited capacity of transvascular leakage of large drug molecules (like vincristine) through the BBB and the increased expression of proteins responsible for the drug efflux system, such as p-glycoprotein, in the BBB [49,50]. Due to the systemic side effects of high doses of drugs and insufficient concentration in the brain tissue, various methods have been tried to increase the effectiveness of current chemotherapy. For instance, osmotic destruction of the BBB using mannitol or modification of the BBB permeability by bradykinin or its analogues were studied. The first method increases the influx of other molecules into the brain, such as albumin, which can lead to edema. Bradykinin and its analogues are more selective. However, despite the benefits of the analogue (RMP-7) obtained in Phase II clinical trials in patients with malignant glioma, the efficacy of these substances in Phase III has not been demonstrated [3,51]. Other options include the use of ultrasound or radiation therapy to improve the penetration of the substance through the BBB [52]. Table 2 shows the various possibilities for modulating BBB permeability, including pharmacological and physical methods. Another mode of drug administration is direct implantation into the tumor of a carrier that carries the drug that is gradually released, e.g., a carmustine/temozolomide wafer implant [53]. The method enables the substance to act directly on neoplastic cells, simultaneously reducing systemic effects. However, the invasiveness of the implantation process can be associated with adverse events like surgical site infection, acute hematoma, wound healing complications, or mass effect [54]. The data presented above show a strong need for further development of already available therapies and searching for innovative methods of treating primary brain tumors. 

## 4. Adenosine Receptors

### 4.1. Characteristics of Adenosine Receptors

There are 4 types of adenosine receptors. A2A and A2B interact with the G_s_ protein to activate adenylate cyclase, while A1 and A3 interact with the G_i/0_ proteins, which reduce the activity of adenylate cyclase [72]. The affinity of A2A receptors for G_s_ proteins depends on the β subunit; this interaction is the strongest with the G proteins containing the β4 subunit. Ultimately, activation of the receptor leads to an increase in the concentration of cAMP in the cell. The expression of A2A receptor is regulated by protein kinase C and altered under pathological conditions. Using radiological techniques, A2A receptors were located on leukocytes (e.g., neutrophils), platelets, blood vessels, and also within the CNS, e.g., in the striatum [73]. These receptors are activated primarily by adenosine but also by inosine and other exogenous molecules used in therapy [74,75]. In pathological conditions, the expression of proteins affecting BBB function may be altered. The expression of the A2A receptor in glial cells is increased in the presence of hypoxia or inflammation by the action of factors such as interleukin-β or tumor necrosis factor α [76].

### 4.2. Function and Mechanism of Action of A2A Receptors

Ledent et al. proved that the activation of A2A receptors inhibits the aggregation of platelets and regulates blood pressure through vasodilation. A2AR agonists also modulate the pain pathways [77]. The vasodilating effect of A2A receptor agonists were demonstrated in the coronary arteries of rats as well as the mesentery of dogs. Gong Zhao et al., showed that vascular resistance-reducing effect of the CVT-3146 adenosine receptor agonist was dose dependent and had more potent influence on coronary arteries than adenosine [78,79]. 

Another important function of A2A receptor agonists is to protect the tissue from damage by reducing inflammation (affecting neutrophils, platelets, macrophages, and T cells) during ischemic reperfusion, e.g., in hemorrhagic shock [80,81]. Moreover, adenosine agonists in the CNS alleviate inflammatory processes and show a protective effect on nerve cells [82].

**Figure 2:**
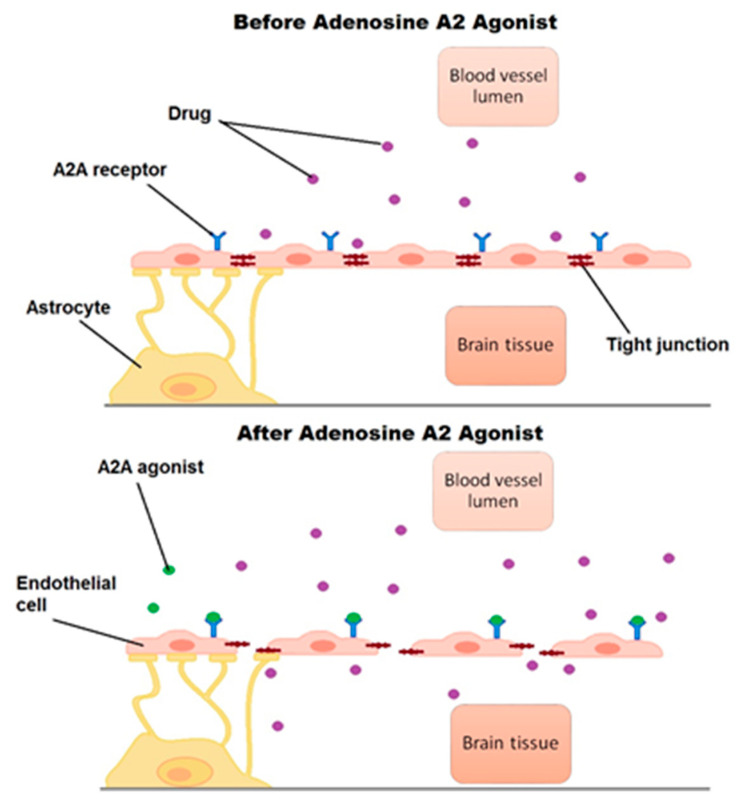
Effect of adenosine receptor agonists on the permeability of the blood–brain barrier.

Adenosine 2A agonists increase the permeability of the blood–brain barrier by actin-cytoskeletal reorganization, which is an indispensable part of the intercellular junction system (Figure 2). Several different mechanisms are involved in altering the function of tight and adhesive junctions: increase in RhoA signaling activity and formation of actin stress fiber, downregulation of VE-Cadherin, ZO-1, and Claudin-5, and reduced phosphorylation of adhesion-related factors. These alternations lead to the enhancement of the paracellular gap and increased leakage of substances through the BBB [62,76,83].

Hurtado-Alvarado et al. noticed that sleep restriction leads to increased inflammatory response and changes the blood–brain barrier function. The processes were related to the influence of adenosine on receptors in the vascular endothelium of the brain. Sleep restriction increased the permeability of the blood–brain barrier as indicated by the increased transport of FITC dextran and Evans blue to the brain. The expression of the adenosine receptor in the basal ganglia and the hippocampus were also increased. Reduction in the expression of tight junction proteins (such as claudin-5, occludin, and ZO-1) and the adherence junction (e-cadherin) occurred in the experiments as well. Subsequently, the effects of the A2A receptor antagonist (SCH58261) were also examined. The agonist reversed the effect of sleep restriction, confirming the participation of adenosine receptors in the processes [84]. 

Another proposed mechanism of A2AR agonists action is the reduction of P-glycoprotein (P-gp) expression on endothelial cells in the CNS. The role of P-glycoprotein is to remove foreign substances, such as drugs, from the inside of the cells, preventing them from accumulation and making it harder to reach their destinations. In an in vitro study, Lexiscan reduced expression of P-gp resulting in an accumulation of the chemotherapeutic drug in brains of mice and human brain endothelial cells [85].

### 4.3. A2AR Agonists

Various adenosine A2 receptor agonists were synthesized, but only a few have been tested for their effects on blood–brain barrier function. Those substances differ in their selectivity to A2A receptors and duration of action. The adenosine 2A receptor activating molecules include [78,79,86]:

low affinity, short-acting, and selective: e.g., CVT-3146 (Regadenoson) and CVT-3033;
high affinity, longer duration of action, and selective: e.g., CGS21680, ALT-146e (Apadenoson), ALT-313 (Evodenoson), or WRC0470;
nonselective agonists: e.g., NECA (5′-*N*-Ethylcarboxamidoadenosine).

Agonists with high affinity for the receptor have a longer duration of action, while for molecules with lower affinity have a shorter time of action. Long-acting agonists may be beneficial for treatment. However, for the radiological imaging of the coronary arteries over time, agonists with lower affinity and shorter duration of action are a more favorable alternative. Importantly, the potency of agonistic action remains the same; the drugs differ only in the time of response reversibility. The disadvantage of high-affinity agonists is the low organ selectivity, which may cause not only increased flow in the coronary vessels but also excessive vasodilatation of the peripheral vascular bed. This causes a great limitation in the use of the agonistic molecules. Compounds with low affinity generally give a weaker response in target tissues. In addition, short-lasting agonists have less impact on other organs, giving less side effects. Moreover, with lower affinity of the agonists toward the receptors, the potency of the action is more dependent on the expression of the adenosine A2 receptor within the targeted tissues. This may contribute to higher tissue selectivity. Therefore, it is important to determine the level of receptor expression in the target tissues to access whether a given substance would have therapeutic or diagnostic efficacy [78]. 

Several attempts were made to use certain molecules to change the permeability of the blood–brain barrier. Table 3 presents the examples of adenosine receptor agonists with a proven effect on altering the permeability of the CNS barrier include CVT-3146 (also known as Regadenoson or Lexiscan—FDA approved agonist), NECA, and CGS 21680.

In Kim and Bynoe studies, Lexiscan was intravenously administered with 10 kDa FITC-Dextran. It has been shown, that Lexiscan at a dose of 0.05 mg/kg increases the permeability of the BBB in mice. BBB permeabilization is time dependent and reversible [62]. Similar results were obtained in studies with the use of A2R agonists—NECA by Carman A.J. et al. [83]. Compared to Lexiscan, NECA induced more gradual change in the BBB permeability, with the greatest effect observed after 6–7 h (by contrast—Lexiscan after 30 min).

Recently, attempts were made to use nanotherapy in treatment of brain tumors. Due to the material, nanoparticles (NPs) can be divided into organic and inorganic. They vary in size and show different mechanical, optical, and electrical properties. Main functions include targeted drug delivery and drug release, tissue imaging, and photothermal and photodynamic therapy. Table 4 shows the characteristics of NPs and their main medical function [65,88,89,90].

NPs can also exhibit theranostic properties. At the same time, they enable diagnostic and therapeutic applications. Magnetic or gold NPs deliver anticancer drugs to brain tissue while allowing tumor visualization with imaging techniques such as MRI or CT [91,92]. Moreover, gold NPs have radiosensitizing properties and may be useful in combination with radiotherapy [93]. Another advantage of nanotechnology is the ability to attach tumor-specific ligands to nanomaterials, increasing their selectivity for tumor cells. Hydrophobic drug conjugated with targeted gold NPs showed 10 fold improved selectivity to brain tumor cells than untargeted conjugates [94]. However, despite its many advantages, nanotechnology is a new multifunctional system in medicine, and the long-term side effects are not fully understood. Side effects such as potential neurotoxicity, systemic toxicity, or the risk of accumulation of nanomaterials in various tissues should be thoroughly explained [95]. It is worth mentioning that despite the many advantages of this group of molecules, individual NPs have only some of them. For instance, liposomes are characterized by high biocompatibility and low toxicity, but their diameter reaches even 1000 nm. Additionally, only particles with ferromagnetic properties or containing metals can be simultaneously used in tissue imaging as a contrast agent [96]. NPs smaller than 12 nanometers (e.g., some dendrimers) can reach therapeutic concentrations in individual cells of the CNS. Larger molecules exceed the pore size of the BBB and therefore are not able to accumulate in brain tissue at a therapeutic concentration [97]. Otherwise, when cancer cells infiltrate adjacent tissues extensively and the BBB is disrupted, NPs can reach the tumor cells and release the encapsulated drug directly to the tumor area. However, as previously mentioned, the BBB is often only partially altered in the tumor area and an intact BBB prevents the extravasation of nanoparticles [98]. In this case, conjugation of A2R agonists to NPs may enhance the concentration of drugs at the target site. Importantly, available A2AR agonists have a short circulating lifetime (up to 5 min) in mammals [99]. Combination of these activators with ligand-functionalized NPs enhances selectivity, improves targeted drug delivery in tumor cell targeting, and prolongs the time-window of the BBB opening, which maximizes the effect of adenosine agonists and antitumor activity of drugs [64,99]. NPs containing A2AR agonists have been shown to increase an uptake of the 45 kDa model drug in the CNS [100]. Currently, the most commonly used A2AR agonist in nanoparticles is Lexiscan, but NECA has also shown a beneficial effect in increasing the BBB permeability by acting on TJs of endothelial cells [101]. Beneficial effects were obtained in the treatment of ischemic stroke, limiting infarct volume in the brains of mice [102]. Nanoparticle drug delivery with doxorubicin and lexiscan was also more effective in treating the orthotopic human U87MG glioblastoma multiforme and breast cancer brain metastases in murine models [64,103]. Enhanced blood circulation time, improvement in BBB penetration, inhibition of tumor growth, and prolonged survival were observed [64]. Other in vivo studies on mouse brain glioma cells showed the improved antitumor activity and increased survival rate in mice treated with a combination of chemotherapy (paclitaxel imaging injection) with an A2A nanoagonist compared to chemotherapy alone. The A2A receptor nanoagonist reduced the expression of ZO-1, the TJ-forming protein, while the formation of actin stress fibers leading to the contraction of cells followed by TJ disrupture was significantly increased. The combined treatment improved (*p* ≤ 0.01) the maximal survival time from 32 to 40 days after tumor implantation. A 2.9-fold delay in tumor growth was observed in group treated with nanoagonist plus PTX, compared to treatment with PTX alone [15].

### 4.4. Clinical Implication and Uncertainties

Lexiscan (regadenoson) is already used in cardiology for pharmacological exercise testing, due to its properties that increase coronary blood flow. The bolus injection of Lexiscan in dose 0.4 mg/5 mL over 10 s followed by injection of the radioactive tracer enables radionuclide imaging of myocardial perfusion in patients for whom exercise testing is impossible [104]. However, the presence of side effects such as headache, dizziness, diarrhea, and abdominal discomfort were noticed [105,106]. Despite the effectiveness of Lexiscan in altering the BBB permeability in mice, similar results were not obtained in a pilot study with the administration of Lexiscan at doses approved by the FDA in humans. In a clinical study by Suhan et al., patients with indications for pharmacologic cardiac stress testing received radiotracer alone for rest cardiac imaging and radiotracer with Lexiscan (bolus injection of 0.4 mg Regadenoson) for stress cardiac testing. In addition to cardiac imaging, imaging of the brain (SPECT or CT) at various time points after radiotracer injection was performed. No significant difference in mean brain uptake of imaging agents after Lexiscan administration was observed [107]. It should therefore be assessed whether a different A2AR agonist or a higher dose of Lexiscan would be effective in altering blood–brain barrier permeability in humans. A clinical trial (ClinicalTrials.gov (accessed on 29 March 2021), number: NCT03971734) is currently underway to determine a safe regadenoson dose that transiently affects the integrity of the blood–brain barrier in patients with high-grade gliomas. Notably, 45 participants of the study will be divided into 7 groups and receive Regadenoson in a dose of 0.05–1.4 mg. Subsequently, 10 min after injection of adenosine agonist, an MRI will be performed to assess the concentration of gadolinium in the CNS [108].

To assess the possibility of clinical use of Lexiscan in the treatment of brain tumors, it is necessary to consider not only the dosage but also the possible protocol of administration of this drug. For instance, the time gap between Lexiscan and antitumor drug administration is of great importance. Furthermore, to prevent accumulation and side effects, a number of doses and the time interval between consecutive doses should be considered. Due to the current use of Regadenoson in cardiology, the pharmacokinetics and pharmacodynamics of this substance in human have already been studied. It has been shown that the standard dose (0.4 mg) sufficiently increases the coronary blood flow (two-fold increase) for approximately 9 min. Re-administration of Regadenoson 10 min after the first injection results in an excessive increase in mean plasma concentrations, which enhances the risk of side effects. Taking into account the half-life and the time of the clinical effect of Regadenoson, the interval between successive doses should be extended to about 150 min [109,110]. In turn, analyzing the proper time gap between Lexiscan and anticancer drug injection, it should be seen that the BBB opening time window after Lexiscan administration is up to 50 min, while after the use of nanoagonists, it is possible to extend this period up to 2 h [99]. This property may be beneficial in adjusting the interval between administration of Lexiscan and the anticancer drug depending on the pharmacokinetics of the therapeutic drug.

Another important aspect is the use of such a technique that allows for the modulation of the BBB permeability by activation of A2A receptors in the CNS without affecting peripheral receptors. This action reduces systemic side effects, including vasodilation, dizziness, or headaches. One of possible methods might be intra-arterial administration of drugs, e.g., into the carotid artery. The effectiveness of this method in modulating BBB has already been proven for the agonists of the bradykinin B2 receptor—RMP-7 [111]. In turn, the intrathecal injection of an adenosine agonist (A2 agonist-CPCA) influenced the cardiovascular system, causing a decrease of blood pressure and heart rate in rats [112]. A promising direction seems to be the synthesis of nanoparticles functionalized with ligands, e.g., antibodies. These ligands, by binding specifically to receptors in cerebral vessels or expressed by tumor cells, would limit the systemic effects of the drugs. Interestingly, great potential is also presented by the targeted drug release mechanism, in which the use of, e.g., UV light causes the encapsulation of drugs at the target site [113].

Recently, Yan A. et al. showed that the increased activity of the adenosine producing enzyme (CD73) is associated with the progression of glioblastoma. An experiment using mouse models showed that the presence of CD73 is associated with a larger tumor size, invasiveness of glioblastoma cells, and the promotion of angiogenesis in comparison to the cells that do not express CD73 protein. Moreover, blockade of the A2B receptor has been shown to promote tumor cell death during temozolomide therapy by reducing both, permeability glycoprotein (P-GP) expression and drug resistance [114]. Other studies also confirm the role of adenosine in the progression of brain tumors, mainly through the activation of A1, A2B, and A3 receptors [115,116,117]. Lexiscan led to the reduction in P-GP protein expression, which resulted in a better response to chemotherapy (with temozolomide). Interestingly, some studies demonstrated an increase in multidrug resistance after exposure to adenosine, which stays in contrast with the previously mentioned mechanism of A2A agonist’s action [85,118]. Probably, the selectivity of the substance toward the A2A receptor is of greatest importance in this case. However, before adenosine agonists would be used in the treatment, the remaining unclarities should be thoroughly addressed.

## 5. Summary

The BBB, despite its invaluable protective role, is a major obstacle in the therapy of primary brain tumors. Currently available treatments for glioblastoma multiform are insufficient. The average survival rate for this type of tumor is around 12 months. Adenosine 2A receptor modulation may be a potential target to increase the effectivity of chemotherapeutics and to improve the results of cancer therapy.

The modern nanomedicine-based drug delivery system enables the drug to enter the tumor area and more effectively act on cancer cells. Nanoparticles containing A2A receptor agonists seem to be a promising direction in research aimed at improving the treatment of brain tumors, as well as other CNS diseases.

When exploring or designing novel compounds, the researchers should focus on minimizing systemic side effects and strive for the greatest selectivity of the agonist to the A2A receptor. Further research is required to determine the appropriate dose of the drug, dosing schedule, and possible side effects, which would allow for simultaneously safe and effective treatment of patients with malignant brain tumors.

## Figures and Tables

**Table 1:** Characteristics of drugs used in the treatment of CNS malignant tumors, including pharmacokinetics, penetration through the BBB, and systemic side effects.

Drug	Pharmacokinetics and BBB Penetration	Systemic Side Effects	References
Carmustine	Partial BBB penetration—local application can bypass both the short serum half-life and the systemic toxicity	Bone marrow suppression, pulmonary fibrosis—systemic delivery is not associated with a significant prolongation of the patient’s survival	[23,24]
Lomustine	Partial BBB penetration—oral administration, rapid metabolism, and lipophilicity	Myelosuppression, nausea, fatigue, and pulmonary fibrosis	[25,26,27]
Irinotecan	Under investigations, crosses the BBB, works in mono- and poly-chemotherapy against brain tumor xenografts and MDR glioblastoma cells	Myelosuppression, neutropenia, gastrointestinal toxicity, nausea, vomiting, and diarrhea	[28,29]
Melphalan	In rat model, it is transported actively by large amino acid transporter and in high (>1 mM) concentrations may open the BBB in a nonspecific manner.	Suppression of ovarian function, amenorrhea, azoospermia, reversible and irreversible testicular suppression, embryo lethality, malformations, predisposition to pneumonitis and gastrointestinal toxicity, marrow aplasia, cardiac dysrhythmia, hemorrhagic diarrhea, and bowel perforation	[30,31]
Methotrexate	For CNS lymphomas treatment, high-dose intravenous MTX penetrates the BBB, effective in combination with BBB disrupting agents	Gastrointestinal tract symptoms, myelosuppression, pancytopenia, liver dysfunction, renal failure, pulmonary symptoms, and mucositis, ulcerations	[32,33]
Procarbazine	Readily cross of BBB, rapid equilibration between plasma and CSF	Gastrointestinal disturbances, myelosuppression, hair loss, fever, chills, generalized aches and pains, weakness, lack of balance, headache, dizziness, rash, neutropenia and thrombocytopenia, myalgia, and arthralgia	[34,35]
Temozolomid	Can penetrate BBB due to its lipophilic structure and small size of the molecule	Vomiting, nausea, constipation, tiredness, dizziness, and anorexia	[36,37,38]
Thalidomide	Penetrates BBB, modulates BBB glial cells	Birth defects, peripheral neuropathy, rash, fatigue, constipation, thrombosis, Stevens–Johnson syndrome, malaise, and edema	[39,40,41]
Thiotepa	Thiotepa and its active metabolite, tepa, efficiently cross the BBB	Cognitive impairment, nausea, vomiting, hair loss, pain sores, bleedings, rashes, and dermatitis	[42,43]
Vincristine	The lipophilic agent penetrates BBB when supplied intravenously	Blurred vision, walking difficulties, jaw pain, numbness, pain in the extremities, stomach cramps, neurotoxicity, effect on seminiferous tubules, cardiovascular disorders, alopecia, rash, coma, and paralysis	[44,45,46,47]

**Table 2:** Methods for modulating BBB permeability including challenges, common side effects, and clinical phase progress of these methods.

Molecules Affecting BBB Used in Treatment of CNS Diseases	Effect on BBB	Challenges and Side Effects	Example of Substance	Clinical Phase Progress	References
Pharmacological					
Phosphodiesterase 5 (PDE5) inhibitors	Increased permeability of brain capillaries by inhibition the degradation of cGMP and increased vesicular transport in tumor area	Headache, flushing, dyspepsia, nasal congestion, nasopharyngitis, and visual abnormalities.	Sildenafil (Viagra) Vardenafil (Levitra)	Preclinical	[55,56]
Potassium channel activators	Selectively increases BBB permeability in the tumor area via a transcellular pathway and downregulation of the expression of tight junction proteins, increased formation of pinocytotic vesicles	pericardial effusion, cardiac tamponade, reflex tachycardia, hypotension, dermatologic reactions, and hypertrichosis	Minoxidil sulfate	Preclinical	[57,58]
Bradykinin receptor activators	Selectively and temporarily increased tumor BBB permeability—multidirectional effect (increased transcytosis, modulation of TJ proteins and cGMP synthesis)	A short biological half-life, in phase III clinical trials the efficacy of RMP-7 has not been confirmed. Side effects: flushing, nausea, headache, and increase in heart rate	Labradimil (reffered to as RMP-7)	Clinical	[3,59,60]
Osmotic substances	Osmotic disruption of BBB, efficacy proven in preclinical and clinical studies	Enhanced entry of other molecules such as albumin to CNS. Side effects: edema, seizures, or neuropathological changes	Mannitol/arabinose	Clinical	[51,61]
A2AR agonist	Effective increase in BBB permeability in mice and rats via downregulation of the expression of tight junction proteins and P-glycoprotein	Short circulating lifetime, systemic side effect, no efficacy in clinical trial in FDA approved doses	Lexiscan	Clinical	[62,63]
A2AR agonist + nanoparticles	As above, but comparing to A2AR agonist alone: enhanced selectivity (may correlate with reduced systemic side effect), improved targeted drug delivery to CNS, prolonged time window of the BBB opening	Effectiveness proved only in in vivo studies—no clinical trials	NPs with Lexiscan	Preclinical	[64,65]
Physical					
Stereotactic or Microbeam Radiation Therapy (MRT)	Increase in tumor vessel permeability in rats after irradiation	Early radiation toxicity syndrome, inhibition of cell regeneration, demyelination, and tissue necrosis possible	-	Preclinical	[52,66,67,68]
Focused Ultrasound (FUS)	Downregulation of TJ proteins induced transcellular transport—increased number of transport vesicles	Risks associated with over-activation of the immune system, such as autoimmunity, vascular damage due to microbubble inertial cavitation when using intensive FUS parameters	-	Clinical- phase 1 clinical trial	[69,70]
Laser-Induced Thermal Therapy (LITT)	Increased BBB permeability in patients with the highest permeability observed 1–2 weeks after thermal ablation	-invasive -general anesthesia required	-	Clinical	[71]

**Table 3:** Examples of A2A receptor agonists with a proven effect on altering the permeability of the BBB.

Adenosine 2A Agonist	Mechanism of Action	Clinical Application	Research Object/Model	Effect on BBB Permeability	References
Regadenoson (CVT-3146/ Lexiscan)	Selective adenosine 2A receptor agonist	FDA approval for pharmacologic cardiac stress testing (detection of coronary artery disease)	In vitro: primary human brain endothelial cell monolayers	Increase in BBB permeability for 10 kDa FITC-dextran mainly from 5 to 30 min after administration	[62]
			In vivo: murine models	Increased permeability of the BBB to 10 kDa FITC-dextran (maximal concentration after 30 min)	[62]
			In vivo: -murine models -rat models	Increased CNS dextran entry over time (maximum after 30 min) in both mice and rats	[83]
			Clinical study—patients with glioblastoma	No significant difference in TMZ concentrations in CNS before and after administration of Lexiscan	[63]
			In vivo: -murine models -rat models	Significantly increased concentration of voltage sensitive dye (VSD) in rat brain tissue and increased residence time of the VDS fluorescence signal in mouse brains.	[87]
			In vivo: rat models	Significantly higher brain temozolomide concentrations at 120 min after regadenoson and TMZ administration	[48]
NECA (5‘-N-Ethylcarboxamidoadenosine)	Broad-spectrum adenosine receptor agonist	Not yet approved by the FDA	In vitro: -primary human brain endothelial cell monolayers	Increase in BBB permeability for 10 kDa FITC-dextran mainly from 60 to 90 min after administration	[62]
			In vivo: -murine models -rat models	Increased entry of 10 kDa and 70 kDa dextrans into WT mouse brain 3 h after intravenous administration	[83]
CGS 21680	Selective adenosine 2A receptor agonist	Not yet approved by the FDA	In vivo: -murine models	Increased entry of 10 kDa FITC-dextran into WT brain tissue 3 h after intravenous administration	[83]

**Table 4:** Characteristics of NPs and their main functions in the treatment of malignant brain tumors.

Nanoparticles	Examples	Main Medical Applications	Additional Properties	Challenges
Organic	Dendrimers	-drug delivery system to CNS -extended circulation time of drugs -targeted drug release -reduced toxicity of anticancer drugs		-only small-size NPs can cross BBB ( <12 nm) -not fully explored, further research required -potential neurotoxicity and systemic toxicity
	Liposomes		-sensitivity to light	
	Micelles			
	Polymeric NPs			
Inorganic	Gold NPs	-drug delivery system to CNS -extended circulation time of drugs -targeted drug release -reduced toxicity of anticancer drugs -tissue imaging	-photothermal therapy -enhanced sensitivity to radiation—combined therapy possible	
	Silver NPs			
	Iron oxide NPs		-photothermal therapy	
	Silica NPs			
	Quantum Dots		-extremely small size 2–20 nm	
